# The association of cardiovascular risk factors and depressive symptoms on cognitive function in the U.S. POINTER study

**DOI:** 10.1002/alz.087549

**Published:** 2025-01-03

**Authors:** Amber A Thro, Katelyn R Garcia, Kristin R Krueger, Marjorie Howard, Heather M Snyder, Laura D Baker, Kathryn V Papp

**Affiliations:** ^1^ Wake Forest University School of Medicine, Winston‐Salem, NC USA; ^2^ Rush University Medical Center, Chicago, IL USA; ^3^ Alzheimer’s Association, Chicago, IL USA; ^4^ Wake Forest University, Winston‐Salem, NC USA; ^5^ Brigham and Women’s Hospital, Boston, MA USA

## Abstract

**Background:**

Cardiovascular risk factors and depressive symptoms have both been independently shown to be negatively associated with cognitive function. However, the nature of the influence of comorbid depressive symptoms and cardiovascular risk on cognitive function is unclear, and there have been inconsistent findings as to which cognitive domains may be most associated with this relationship.

**Method:**

U.S. POINTER is a randomized controlled trial of two multidomain lifestyle interventions. We examined the baseline relationship between cardiovascular risk, depressive symptoms, and cognitive function in 2103 U.S. POINTER participants (mean age = 68.19±5.16y). The Framingham Risk Score (FRS) using lipids was used to calculate the 10‐year risk of developing cardiovascular disease, and depressive symptoms were assessed using the 15‐item Geriatric Depression Scale (GDS). Cognitive function was measured using composite scores (global, episodic memory, executive function, and processing speed) derived from the U.S. POINTER modified Neuropsychological Test Battery. After computing correlations, generalized linear regression models were used to determine the presence of an interaction between GDS (using a median split of 0‐1 and 2+) and FRS for each cognitive composite while controlling for demographic variables.

**Result:**

Higher GDS was significantly associated with lower global cognition (r = ‐0.04, p = 0.04) and slower processing speed (r = ‐0.06, p = 0.01) but not episodic memory (r = ‐0.03, p = 0.17) or executive function (r = ‐0.02, p = 0.37). FRS was negatively associated with global cognition (r = ‐0.25, p<0.01), episodic memory (r = ‐0.27, p<0.01), executive function (r = ‐0.13, p<0.01), and processing speed (r = ‐0.20, p<0.01). Regression models identified the presence of an interaction between FRS and GDS with global cognition (p<0.01). Specifically, participants with lower GDS scores (0‐1) had higher global cognition at lower levels of FRS than those with higher GDS scores (2+), but as FRS approached 16%, participants with lower GDS scores had lower global cognition than those with higher GDS scores. The presence of an interaction between FRS and GDS was also found with the executive function (p<0.01) and processing speed (p = 0.04) composites, but not with the episodic memory (p = 0.12) composite.

**Conclusion:**

In U.S. POINTER participants at baseline, the effect of cardiovascular risk on global cognition, executive function, and processing speed may vary depending on the presence and severity of depressive symptoms.

